# Increased anterior insula connectivity associated with cognitive maintenance in amnestic mild cognitive impairment: a longitudinal study

**DOI:** 10.1007/s11682-024-00899-2

**Published:** 2024-05-24

**Authors:** Hui Li, Xiang Fan, Kuncheng Li, Chen Zhang, Xiuqin Jia

**Affiliations:** 1 Department of Radiology, Beijing Chaoyang Hospital, Capital Medical University, No. 8, Gongti South Road, Chaoyang District, Beijing, 100020 China; 2 Department of Medical Imaging, Peking University Shenzhen Hospital, Shenzhen, 518036 China; 3 Department of Radiology, Xuanwu Hospital, Capital Medical University, Beijing, 10053 China; 4 MR Research Collaboration, Siemens Healthineers, Beijing, 100102 China

**Keywords:** Amnestic mild cognitive impairment, Insula subregions, Resting-state functional connectivity, Longitudinal study

## Abstract

The insula, a crucial hub of the human brain network, can be divided into anterior and posterior regions. Previous studies have reported that different insula subregions play various roles in amnestic mild cognitive impairment (aMCI). However, the longitudinal changes in the functional connectivity (FC) of each insula subregion in aMCI patients over time remain unclear. Twenty aMCI patients and 20 healthy controls (HCs) were recruited and underwent resting-state functional magnetic resonance imaging (fMRI) scans and neuropsychological assessments at baseline and at the 15-month follow-up. FMRI data were preprocessed using SPM 12 and the CONN toolbox. Two-way analysis of covariance was used to compare longitudinal changes in the FC of each insula subregion with covariates including sex, age, education, follow-up interval, volume of gray matter, and global correlation (GCOR). Pearson’s correlation was used to evaluate the relationship between insula subregional FC and neuropsychological performance in aMCI patients. In aMCI patients, the right anterior insula exhibited significantly increased FC with the left anterior cingulate cortex, whereas the left posterior insula exhibited decreased FC with the right precuneus compared with HCs. Furthermore, FC between the right anterior insula and left anterior cingulate cortex was significantly correlated with global cognition at follow-up. The current findings revealed different functional alterations in the insula subregions and provided new insights into the neurodegenerative process in aMCI patients.

**Supplementary Information:**

The online version contains supplementary material available at 10.1007/s11682-024-00899-2.

## Introduction

Amnestic mild cognitive impairment (aMCI), characterized by cognitive decline without any notable effect on daily activities, is considered the preclinical stage of Alzheimer’s disease (AD) (Petersen et al., 2018). One key feature of aMCI is abnormalities in neural network connectivity between different brain regions, which increases the risk of progression to AD (Biswal et al., 1995; Xie et al., 2012a). To date, researchers have primarily focused on investigating changes in the default mode network (DMN) (Wang et al., 2006). However, recent studies have suggested that the salience network (SN) is another crucial network involved in aMCI and AD (Chand et al., 2017a; Wang et al., 2015). The insula, as a primary anchor point of the SN, also plays a vital role in AD (Liu et al., 2018; Xie et al., 2012a).

Previous studies have shown that the insula is associated with memory encoding (Daselaar et al., 2004) and episodic memory processing (Nellessen et al., 2015). Significantly reduced gray matter volume (Kim et al., 2020), increased activation (Zanchi et al., 2017), increased regional homogeneity (ReHo) (Liu et al., 2014), and decreased synaptic density (Zhang et al., 2023) have been observed in the insula of aMCI patients. Abnormal functional connectivity (FC) in the insula (Xie et al., 2012a) and cerebral glucose metabolism (Seo & Choo, 2016) are associated with clinical cognitive deterioration in aMCI patients. In one study, when switching from a resting to a working memory state, the FC between the insula and angular gyrus increased in healthy controls (HCs), while no significant change was observed in aMCI patients (Wang et al., 2017).

A previous study highlighted that the insula is composed of anterior and posterior regions, divided by the central sulcus (Xie et al., 2012a). Using diffusion tensor imaging (DTI)-based tractography or a data-driven clustering technique, several recent studies mapped the organization of the human insula and the whole-brain FC patterns of different insula subregions (Liu et al., 2018; Deen et al., 2011; Taylor et al., 2009; Xie et al., 2012a). Taylor et al. reported that the anterior insula cortex (AIC) is primarily connected to the dorsal anterior cingulate cortex (ACC), whereas the posterior insula cortex (PIC) is primarily connected to the somatomotor cortex. Furthermore, recent cross-sectional studies reported altered FC of insula subregions and its relationship with cognition in aMCI patients (Liu et al., 2018; Wang et al., 2021). However, no studies have reported longitudinal changes in insula subregional connectivity in patients with aMCI over time.

This study aimed to explore longitudinal changes in insula subregional FC in aMCI patients. We hypothesized that compared with HCs, aMCI patients would exhibit different longitudinal alterations in insular subregional FC, which would be further associated with clinical neuropsychological performance in these patients.

## Methods

### Participants

The Ethics Committee of Beijing Xuanwu Hospital approved the study, and written informed consent was obtained from each study subject.

Twenty newly diagnosed aMCI patients who were not on medication and 20 HCs were recruited from the memory clinic (all subjects were right-handed). At baseline, all the subjects provided a medical history and underwent a physical examination, magnetic resonance imaging (MRI) evaluation, and neuropsychological assessment, including the Mini-Mental State Examination (MMSE), Montreal Cognitive Assessment (MoCA), Clinical Dementia Rating (CDR), and Auditory Verbal Learning Test (AVLT). After nearly fifteen months, all the subjects completed a follow-up MRI and neuropsychological assessments similar to those at baseline.

All aMCI patients met the diagnostic criteria described by the National Institute on Aging and the Alzheimer’s Association (Albert et al., 2011). In addition, a CDR score of 0.5 with a score of at least 0.5 in the memory domain (Petersen et al., 2001) and a visual rating > 1 for medial temporal lobe atrophy on coronal Tl-weighted MR images were needed (Scheltens et al., 1992). HCs were required to meet the following inclusion criteria: (a) no cognitive complaints; (b) no neurological abnormalities on physical examination; (c) no current or previous diagnosis of psychiatric or neurological disorders; (d) no abnormal findings on brain MRI; and (e) a CDR score of 0.

Subjects were excluded if they had a history of stroke, brain trauma, epilepsy, alcoholism, Parkinson’s disease, depression, any other neurological or psychiatric illness, severe visual or hearing loss, or any other major medical illness. Both aMCI patients and HCs were excluded if they had any contraindications for MRI, including claustrophobia, cardiac pacemakers, or metal implants.

### MRI acquisition

All MRI scans were performed on a 3-Tesla Trio scanner (Siemens, Erlangen, Germany). Foam padding was used to limit head motion, and headphones were used to reduce scanner noise. All the subjects were requested to remain still and awake with their eyes closed. Resting-state functional MR images were acquired with an echo-planar imaging sequence with the following parameters: repetition time (TR)/echo time (TE) = 2000 ms/40 ms, flip angle (FA) = 90°, field of view (FOV) = 256 mm × 256 mm, data matrix = 64 × 64, axial slices = 28, slice thickness = 4 mm, gap = 1 mm, bandwidth = 2232 Hz/pixel, and number of repetitions = 239. The 3D T1-weighted images were acquired with a magnetization-prepared rapid gradient echo sequence with the following parameters: TR/TE = 1900 ms/2.2 ms, FA = 9°, bandwidth = 199 Hz/pixel, inversion time (TI) = 900 ms, FOV = 256 mm× 224 mm, data matrix = 256 × 224, sagittal slices = 176, and slice thickness = 1 mm.

### MRI data preprocessing

Resting-state functional MRI data were first preprocessed using Statistical Parametric Mapping (SPM) 12 software (https://www.fil.ion.ucl.ac.uk/spm). The first ten images of each fMRI dataset were discarded to remove any fluctuations in the initial MRI signal. Images of each individual subject were corrected for slice timing and realignment. All subjects included in the present study exhibited head motion less than 1.5 mm in any of the x, y, or z directions; less than 1.5° in any angular dimension; and volume-level mean framewise displacement (FD) less than 0.30 (with a mean FD across all subjects of 0.16 ± 0.08) (Power et al., 2012). The resulting images were normalized to the Montreal Neurological Institute (MNI) space, resampled to 2 mm × 2 mm × 2 mm, and smoothed with a 4-mm full width at half maximum (FWHM) isotropic Gaussian kernel. After smoothing, subsequent preprocessing was conducted using the functional connectivity (CONN) toolbox v17c (Whitfield-Gabrieli & Nieto-Castanon, 2012). The images were bandpass filtered to 0.008–0.09 Hz to reduce the influence of noise. Further denoising steps included regression and linear detrending. Possible contamination from white matter and cerebrospinal fluid (CSF) signals (Behzadi et al., 2007) and six motion parameters and their first-order derivatives were regressed out from the whole-brain images.

### Definition of insula subregions

**Fig. 1:**
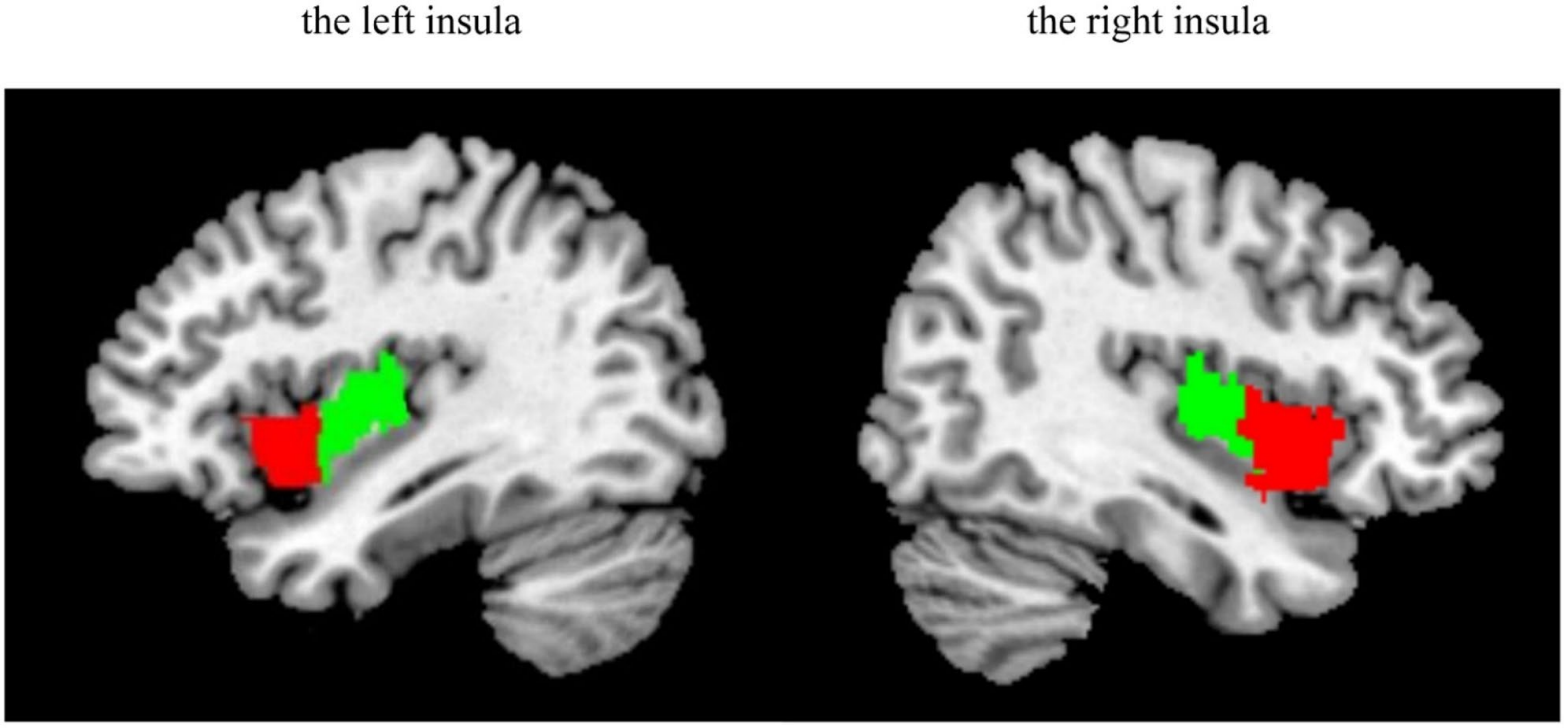
The left and right insula subregions including the anterior insula (red) and posterior insula (green)

Using a data-driven clustering technique, Deen et al. parcellated the human insular lobe into 2, 3, and 4 subregions for each hemisphere, and found that the 2 subregions of insula, anterior and posterior, closely resembled the clustering of DTI-based connectivity patterns (Deen et al., 2011). According to this study, two insular subregions in each hemisphere were defined in the present study. First, the left and right insula were defined anatomically by drawing insular gray matter on the MNI152 standard brain, and whole-brain connectivity maps were computed for each voxel in the insula. After normalization, these resting-state time series were used as regressors in general linear model-based analyses for each subject, and the resulting beta maps were averaged across subjects to obtain the subject-averaged beta map for a given voxel in the insula. Then, these subject-averaged beta maps were treated as vectors, and k-means clustering analysis (k = 2) was applied using squared Euclidean distance as the distance measure, separately for the left and right insula. The k-means algorithm was repeated 100 times, and the solution that minimized within-cluster variance was chosen. Finally, the insula was divided into anterior and posterior subregions (see Fig. 1).

### Gray matter volume analysis

Structural data processing was performed using SPM12, including normalization to the standard MNI space, segmentation, modulation, and smoothing with an 8-mm FWHM Gaussian kernel. The bilateral hippocampi were defined using the Anatomy Toolbox in SPM12. The bilateral hippocampal volumes and insula subregional volumes were then extracted from the smoothed modulated gray matter maps.

### Functional connectivity analysis

Seed-to-voxel correlation analysis was conducted using the CONN toolbox v17c. The mean time series of each insula subregion seed was computed as the reference time course for each individual. Correlation maps were generated by Pearson correlation analysis between each seed time series and those of the other brain voxels. Fisher’s *r*-to-*z* transformation was applied for group analysis to improve the normality of the correlation coefficients (Lowe et al., 1998).

### Statistical analysis

Demographic data and neuropsychological assessments were analyzed using SPSS 22. Student’s t tests were used to compare continuous variables, including age, years of education, neuropsychological assessment scores, and gray matter volumes. Chi-square tests were used to examine differences in sex distribution between the groups.

The patterns of voxelwise insula subregional FC within each group were determined using one sample t tests with no covariates. The results were thresholded at a cluster-level *p* < 0.05 and underwent familywise error (FWE) correction. Then, a 2 (*time*: follow-up *versus* baseline) × 2 (*group*: aMCI *versus* HCs) flexible factorial design was employed, with four conditions, aMCI baseline (aMCI1), aMCI follow-up (aMCI2), HCs baseline (HCs1), and HCs follow-up (HCs2), to detect the main and interaction effects. The covariates used in the between-group analysis included age, sex, educational level, follow-up interval, insula subregion gray matter volume, and global correlation (GCOR) (Saad et al., 2013). GCOR was automatically calculated in the denoising step in CONN. GCOR is the brain-wide average correlation coefficient between every pair of voxels across the entire brain, and it is a conservative approach that can reduce global variations. The results were thresholded at a cluster-level *p* < 0.05 and FWE corrected. The brain regions were visualized with xjView (http://www.alivelearn.net/xjview).

For each insula subregion, the main effects of “*group*” were defined as [(HCs1 + HCs2) vs. (aMCI1 + aMCI2)], and the main effects of “*time*” were defined as [(aMCI1 + HCs1) vs. (aMCI2 + HCs2)]. The “*group*” × “*time*” interaction effects were evaluated for increased FC of the insula subregions in aMCI patients relative to HCs by [(aMCI2 - aMCI1) - (HCs2 - HCs1)] and decreased FC of the insula subregions in aMCI patients relative to HCs by [(aMCI1 - aMCI2) - (HCs1 - HCs2)].

Post hoc pairwise comparisons and correlation analyses were conducted using SPSS 22. The regions showing significant “*group*” × “*time*” interaction effects were defined as regions of interest (ROIs), and the connectivity strength of each ROI was extracted for comparison analysis and correlation analysis. First, independent sample *t* tests were used to compute the group differences in FC between HCs and aMCI patients at baseline and follow-up. Then, paired-sample *t* tests were performed to explore the longitudinal FC changes within each group. Finally, Pearson’s correlations were performed to evaluate (1) the relationship between the FC of each ROI and neuropsychological data in aMCI patients at baseline, (2) the relationship between the FC of each ROI and neuropsychological data in aMCI patients at follow-up, and (3) the relationship between the changes in FC in each ROI and changes in neuropsychological data in aMCI patients over time.

## Results

### Demographic and neuropsychological tests

The demographic data and neuropsychological scores of all the subjects are shown in Table 1. There were no significant differences in age, sex, educational level, or insula subregional volumes between aMCI patients and HCs, whereas significant volumetric differences in the bilateral hippocampi were found. Compared to HCs, aMCI patients showed significant cognitive decline, as revealed by the MMSE, MoCA, AVLT, and CDR scores at baseline and follow-up (*p* < 0.001 for all comparisons). As the disease progressed, more severe memory impairment was confirmed by a lower cognitive level at follow-up than at baseline, as assessed by the MMSE (*p* < 0.017) and MoCA (*p* < 0.048) scores. Two aMCI subjects converted to mild AD, whereas the other aMCI subjects and all the HCs remained stable (Table 1).

**Table 1:** Clinical characteristics of aMCI patients and HCs

	Baseline			Follow-up		
	HCs	aMCI	*p* value	HCs	aMCI	*p* value
Age (years)	67.25 ± 7.50	66.95 ± 9.65	0.91	68.67 ± 7.54	68.10 ± 9.67	0.84
Sex (M/F)	10/10	7/13	0.34	10/10	7/13	0.34
Follow-up interval (months)	17.0 ± 6.5	13.8 ± 3.4	0.06			
Education (years)	11.90 ± 3.82	9.65 ± 4.06	0.08	11.90 ± 3.82	9.65 ± 4.06	0.08
Left hippocampal volume	0.562 ± 0.016	0.538 ± 0.026	0.002	0.560 ± 0.017	0.534 ± 0.027†	0.001
Right hippocampal volume	0.538 ± 0.016	0.521 ± 0.027	0.020	0.535 ± 0.016†	0.516 ± 0.029†	0.013
CDR	0	0.5	< 0.001	0	0.5 (18)/1 (2)	< 0.001
MMSE	28.60 ± 1.23	24.80 ± 3.70	< 0.001	28.35 ± 1.46	23.45 ± 3.47^**†**^	< 0.001
MoCA	26.40 ± 1.63	20.85 ± 4.70	< 0.001	26.50 ± 1.87	19.50 ± 4.91^**†**^	< 0.001
AVLT-immediate recall	8.88 ± 1.63	6.00 ± 1.45	< 0.001	8.95 ± 1.25	6.08 ± 1.38	< 0.001
AVLT-delayed recall	9.35 ± 3.54	4.55 ± 2.78	< 0.001	9.25 ± 3.56	4.90 ± 3.02	< 0.001
AVLT-recognition	11.25 ± 3.05	8.45 ± 3.36	< 0.001	11.70 ± 2.22	7.75 ± 3.24	< 0.001

Note: Values represent the mean ± standard deviation. CDR, Clinical Dementia Rating; MMSE, Mini-Mental State Examination; MoCA, Montreal Cognitive Assessment; AVLT, Auditory Verbal Learning Test. *P* values were derived from comparison of the two groups using Student’s t tests except for “sex”, where the *p* value was obtained using the χ^2^ test. ^**†**^ represents a significant decrease at follow-up vs. baseline in aMCI patients

### Within-group FC of the insula subregions

The within-group FC maps of the insula subregions in HCs and aMCI patients exhibited similar patterns at baseline and follow-up (Supplemental Figures S1-S4). Significant connectivity was identified between the left AIC and the bilateral ACC, paracingulate gyrus, putamen, frontal orbital cortex, inferior frontal gyrus, and temporal pole. The right AIC was positively associated with the bilateral ACC, central opercular cortex, frontal orbital cortex, and right junction region of the temporal, frontal, and parietal lobes. The bilateral PIC exhibited significant connectivity with the bilateral ACC; central opercular cortex; precentral, postcentral, and supramarginal gyri; parietal operculum cortex; and junction region of the temporal, frontal and parietal lobes.

### Between-group differences in longitudinal insula subregional FC changes

No significant differences were found in the main effect of “*group*” or the main effect of “*time*”.

**Fig. 2:**
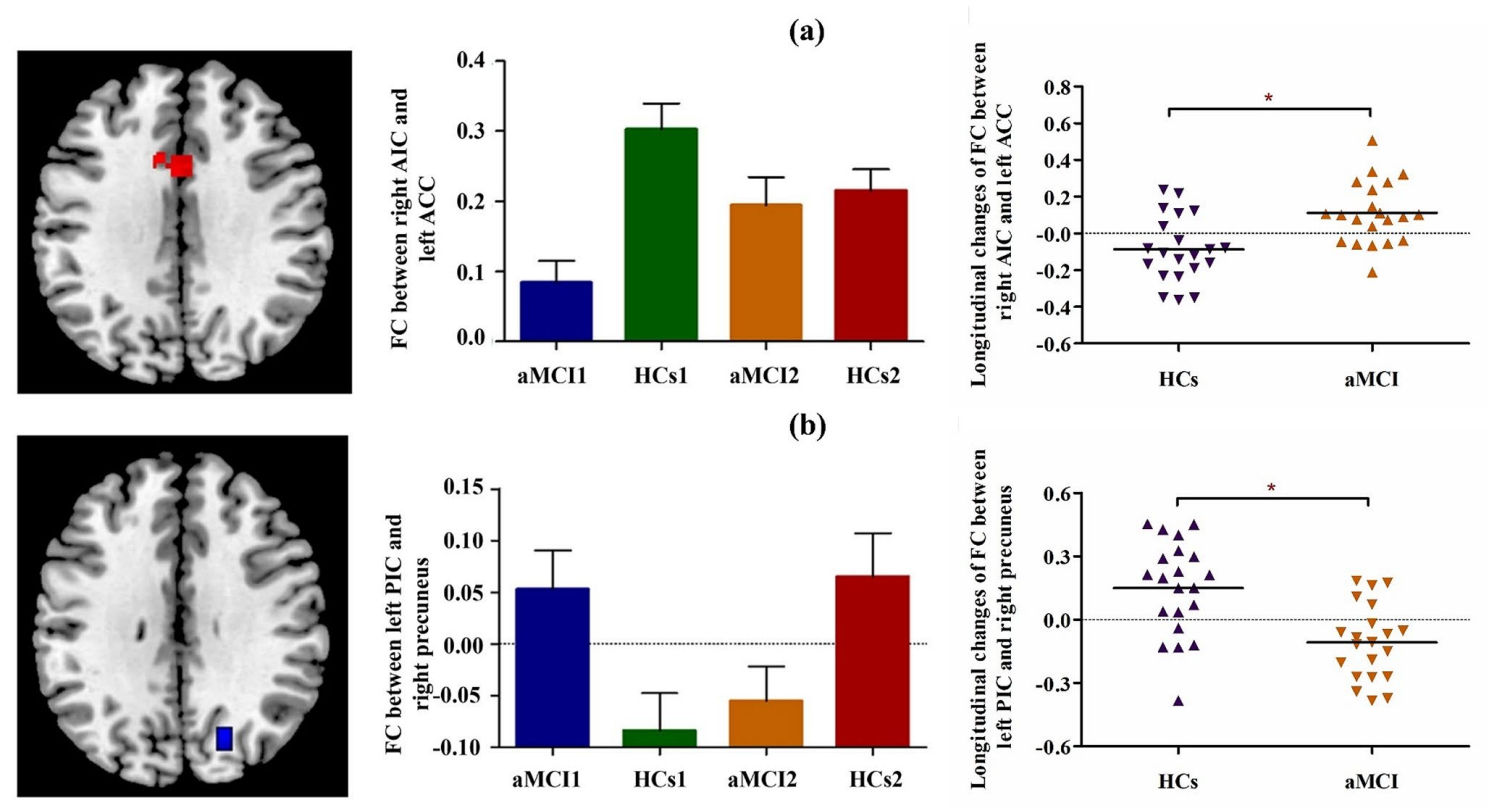
Longitudinal alterations in insula subregional FC in aMCI patients and HCs. (**a**) Increased FC between the right AIC and left ACC in aMCI patients compared to HCs at follow-up vs. baseline (interaction effect). (**b**) Decreased FC between the left PIC and right precuneus in aMCI patients compared to HCs at follow-up vs. baseline (interaction effect). FC, functional connectivity; ACC, anterior cingulate cortex; AIC, anterior insula cortex; PIC, posterior insula cortex

The differences in the longitudinal changes in insula subregional FC between aMCI patients and HCs were explored in the “*group*” × “*time*” interaction effect. Compared with HCs, aMCI patients showed significantly increased FC between the right AIC and left ACC, as well as significantly decreased FC between the left PIC and right precuneus (Table 2; Fig. 2).

**Table 2:** Longitudinal changes in functional connectivity to insula subregions in aMCI patients compared to HCs.

Seed regions	Region	Cluster size (voxel)		MNI (x y z)			T value
Rt.AIC	[(HCs1–HCs2)–(aMCI1–aMCI2)]						
	Lt.ACC		101	-8	14	32	4.08
Lt.PIC	[(aMCI1–aMCI2)–(HCs1–HCs2)]						
	Rt.Precuneus		68	22	-70	32	4.42

Note: Between-group results were thresholded at a cluster-level *p* < 0.05 (FWE corrected). MNI, Montreal Neurological Institute. Rt., right; Lt., left; AIC, anterior insula cortex; PIC, posterior insula cortex; ACC, anterior cingulate cortex

Post hoc pairwise comparisons for FC between the right AIC and the left ACC showed that (1) at baseline, aMCI patients exhibited significantly decreased FC compared with HCs; (2) at follow-up, no significant group differences in FC were found between aMCI patients and HCs; (3) compared with baseline, aMCI patients demonstrated increased FC at follow-up; and (4) compared with baseline, HCs demonstrated decreased FC at follow-up.

Post hoc pairwise comparisons for FC between the left PIC and the right precuneus showed that (1) at baseline, aMCI patients exhibited significantly increased FC compared with HCs; (2) at follow-up, aMCI patients exhibited significantly decreased FC compared with HCs; (3) compared with baseline, aMCI patients demonstrated decreased FC at follow-up; and (4) compared with baseline, HCs demonstrated increased FC at follow-up.

### Relationship between insula subregional FC and cognitive performance

**Fig. 3:**
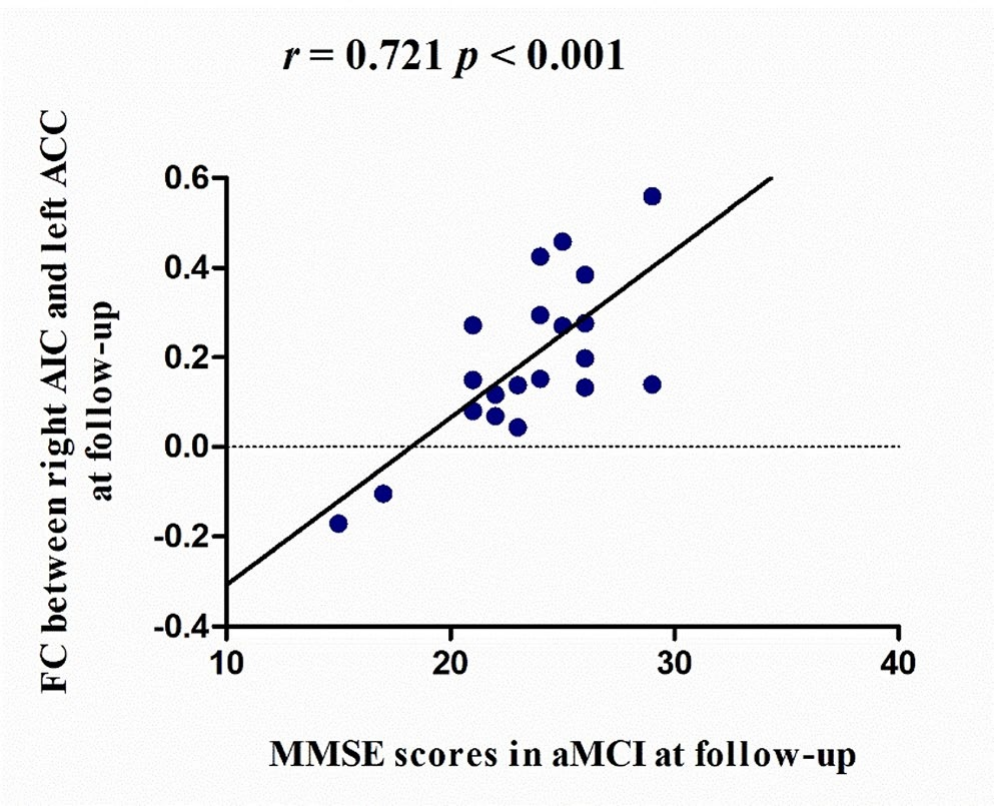
Linear correlation of FC between the right AIC and left ACC and MMSE scores in aMCI patients at follow-up. FC, functional connectivity; ACC, anterior cingulate cortex; AIC, anterior insula connectivity; MMSE, Mini-Mental State Examination

In aMCI patients, Pearson’s correlations were performed between the FC values of the two abovementioned ROIs and the MMSE, MoCA, AVLT-immediate recall, AVLT-delayed recall, and AVLT-recognition scores. These correlation analyses were applied for baseline data, follow-up data, and longitudinal changes. Therefore, statistical significance was set at *p* < 0.05 and Bonferroni corrected for multiple comparisons (*p* < 0.0016, 30 multiple comparisons). In the aMCI group, the FC between the right AIC and left ACC was significantly correlated with MMSE score (*r* = 0.721 *p* < 0.001) at follow-up, suggesting that stronger FC may contribute to the maintenance of cognitive ability (Fig. 3).

## Discussion

The current study demonstrated distinct longitudinal changes in insula subregional FC in aMCI patients by analyzing longitudinal FC data acquired from aMCI patients and HCs. There were two primary findings in aMCI patients compared to HCs over time: the FC between the right AIC and left ACC increased, whereas the FC between the left PIC and right precuneus decreased. In addition, at follow-up, the FC between the right AIC and left ACC was positively associated with global cognition in aMCI patients, as measured by the MMSE score.

A higher level of cognition is attributed to coalition and interaction of different neural networks rather than isolated networks (Xie et al., 2012b). The insula plays a key role in the interaction of sophisticated neural networks. As the disease progresses, aMCI patients may experience different outcomes. Longitudinal studies are more meaningful than cross-sectional studies for investigating the functional neural basis of cognitive deterioration in aMCI patients. The present study revealed that the longitudinal trajectories of insula subregional FC differed between aMCI patients and HCs during follow-up.

Previous studies have described the AIC and ACC as the two most important anchor points of the SN (Menon, 2011). The AIC is responsible for switching between cognitive resources (Touroutoglou et al., 2012), and the ACC is known to be a signal area needed for enhanced cognitive control (Egner, 2009). Unlike disrupted DMN connectivity (Beason-Held, 2011), previous studies reported that SN connectivity was enhanced in patients with AD (Chand et al., 2017b; Zhou et al., 2010). In the present study, the FC between the right AIC and left ACC significantly increased over time in aMCI patients compared to HCs. Furthermore, the FC values were positively associated with the MMSE scores at follow-up. This increased FC between the AIC and ACC might be a compensatory neural mechanism for disease progression and could play a critical role in partially maintaining cognitive function in aMCI patients.

We also found that the FC between the left PIC and right precuneus decreased longitudinally. The precuneus is widely recognized as an essential part of the DMN, which is involved in several cognitive processes (Patel et al., 2015; Zhang & Li, 2012). Furthermore, the precuneus is one of the earliest regions where β-amyloid (Aβ) accumulates (Wolk & Klunk, 2009). Reduced FC of the precuneus has been reported in aMCI patients in previous studies (Binnewijzend et al., 2012; Lee et al., 2016; Qi et al., 2010). Significantly reduced FC between the left PIC and bilateral precuneus was reported in patients with isolated rapid eye movement sleep behavior disorder and was associated with MoCA scores (Byun et al., 2021). The results of the present study showing reduced FC between the left PIC and the right precuneus are consistent with those of previous studies, and this reduced FC may be an underlying mechanism of progressive cognitive decline in aMCI patients.

There were several limitations in this study. First, the sample size was relatively small, and studies with larger sample sizes are needed to replicate the current findings. Second, the mean follow-up interval in aMCI patients and HCs was group-matched, but there were intersubject variations. Although this factor was included as a nuisance covariate in our data analyses, we cannot entirely rule out the influence of the follow-up interval on the current results. Third, without positron emission tomography (PET) examination and CSF analysis, the criteria used in the present study indicated an intermediate likelihood that MCI was due to AD. The A (β-amyloid) – T (tau) – N (neurodegeneration) system should be referred to in future studies (Albert et al., 2011). Finally, resting-state fMRI can be influenced by pulse and breathing. This physiological information should be recorded during scanning and included in data preprocessing to reduce the influence of these variations in future studies.

## Conclusions

The current study revealed different patterns of insula subregional FC changes in aMCI patients over time. These results provide new insights into the neurodegenerative process and may facilitate future research on neural markers of cognitive decline in aMCI patients.

## Electronic supplementary material

Below is the link to the electronic supplementary material.

Supplementary Material 1

## Data Availability

The datasets analyzed in the current study are not publicly available because the project is still in progress. However, the datasets are available from the corresponding author upon reasonable request.

## References

[CR2] Albert, M. S., Dekosky, S. T., Dickson, D., Dubois, B., Feldman, H. H., Fox, N. C., Gamst, A., Holtzman, D. M., Jagust, W. J., Petersen, R. C., Snyder, P. J., Carrillo, M. C., Thies, B., & Phelps, C. H. (2011). The diagnosis of mild cognitive impairment due to Alzheimer’s disease: Recommendations from the National Institute on Aging-Alzheimer’s Association workgroups on diagnostic guidelines for Alzheimer’s disease. *Alzheimers Dement*, *7*(3), 270–279. 10.1016/j.jalz.2011.03.008. 21514249 10.1016/j.jalz.2011.03.008 PMC3312027

[CR3] Beason-Held, L. L. (2011). Dementia and the default mode. *Current Alzheimer Research*, *8*(4), 361–365. 10.2174/156720511795745294. 21222595 10.2174/156720511795745294 PMC3134578

[CR4] Behzadi, Y., Restom, K., Liau, J., & Liu, T. T. (2007). A component based noise correction method (CompCor) for BOLD and perfusion based fMRI. *Neuroimage*, *37*(1), 90–101. 10.1016/j.neuroimage.2007.04.042. 17560126 10.1016/j.neuroimage.2007.04.042 PMC2214855

[CR5] Binnewijzend, M. A., Schoonheim, M. M., Sanz-Arigita, E., Wink, A. M., van der Flier, W. M., Tolboom, N., Adriaanse, S. M., Damoiseaux, J. S., Scheltens, P., van Berckel, B. N., & Barkhof, F. (2012). Resting-state fMRI changes in Alzheimer’s disease and mild cognitive impairment. *Neurobiology of Aging*, *33*(9), 2018–2028. 10.1016/j.neurobiolaging.2011.07.003. 21862179 10.1016/j.neurobiolaging.2011.07.003

[CR6] Biswal, B., Yetkin, F. Z., Haughton, V. M., & Hyde, J. S. (1995). Functional connectivity in the motor cortex of resting human brain using echo-planar MRI. *Magnetic Resonance in Medicine*, *34*(4), 537–541. 10.1002/mrm.1910340409. 8524021 10.1002/mrm.1910340409

[CR7] Byun, J. I., Cha, K. S., Kim, M., Lee, W. J., Lee, H. S., Sunwoo, J. S., Shin, J. W., Kim, T. J., Moon, J., Lee, S. T., Jung, K. H., Chu, K., Kim, M. H., Kim, H. J., Shin, W. C., Lee, S. K., & Jung, K. Y. (2021). Altered insular functional connectivity in isolated REM sleep behavior disorder: A data-driven functional MRI study. *Sleep Medicine*, *79*, 88–93. 10.1016/j.sleep.2020.12.038. 33485260 10.1016/j.sleep.2020.12.038

[CR8] Chand, G. B., & Dhamala, M. (2017b). Interactions between the anterior cingulate-insula network and the fronto-parietal network during perceptual decision. *Neuroimage*, *152*, 381–389. 10.1016/j.neuroimage.2017.03.014. 28284798 10.1016/j.neuroimage.2017.03.014

[CR9] Chand, G. B., Wu, J., Hajjar, I., & Qiu, D. (2017a). Interactions of the Salience Network and its subsystems with the default-Mode and the Central-Executive networks in normal aging and mild cognitive impairment. *Brain Connectivity*, *7*(7), 401–412. 10.1089/brain.2017.0509. 28707959 10.1089/brain.2017.0509 PMC5647507

[CR10] Daselaar, S. M., Prince, S. E., & Cabeza, R. (2004). When less means more: Deactivations during encoding that predict subsequent memory. *Neuroimage*, *23*(3), 921–927. 10.1016/j.neuroimage.2004.07.031. 15528092 10.1016/j.neuroimage.2004.07.031

[CR11] Deen, B., Pitskel, N. B., & Pelphrey, K. A. (2011). Three systems of insular functional connectivity identified with cluster analysis. *Cerebral Cortex*, *21*(7), 1498–1506. 10.1093/cercor/bhq186. 21097516 10.1093/cercor/bhq186 PMC3116731

[CR12] Egner, T. (2009). Prefrontal cortex and cognitive control: Motivating functional hierarchies. *Nature Neuroscience*, *12*(7), 821–822. 10.1038/nn0709-821. 19554047 10.1038/nn0709-821

[CR13] Kim, H. J., Lee, J. H., Cheong, E. N., Chung, S. E., Jo, S., Shim, W. H., & Hong, Y. J. (2020). Elucidating the risk factors for progression from amyloid-negative amnestic mild cognitive impairment to Dementia. *Current Alzheimer Research*, *17*(10), 893–903. 10.2174/1567205017666201130094259. 33256581 10.2174/1567205017666201130094259

[CR14] Lee, E. S., Yoo, K., Lee, Y. B., Chung, J., Lim, J. E., Yoon, B., & Jeong, Y. (2016). Default Mode Network Functional Connectivity in Early and late mild cognitive impairment: Results from the Alzheimer’s Disease Neuroimaging Initiative. *Alzheimer Disease and Associated Disorders*, *30*(4), 289–296. 10.1097/WAD.0000000000000143. 26840545 10.1097/WAD.0000000000000143

[CR16] Liu, Z., Wei, W., Bai, L., Dai, R., You, Y., Chen, S., & Tian, J. (2014). Exploring the patterns of acupuncture on mild cognitive impairment patients using regional homogeneity. *PLoS One*, *9*(6), e99335. 10.1371/journal.pone.0099335. 24968124 10.1371/journal.pone.0099335 PMC4072601

[CR15] Liu, X., Chen, X., Zheng, W., Xia, M., Han, Y., Song, H., Li, K., He, Y., & Wang, Z. (2018). Altered functional connectivity of insular subregions in Alzheimer’s Disease. *Frontiers in Aging Neuroscience*, *10*, 107. 10.3389/fnagi.2018.00107. 29695961 10.3389/fnagi.2018.00107 PMC5905235

[CR17] Lowe, M. J., Mock, B. J., & Sorenson, J. A. (1998). Functional connectivity in single and multislice echoplanar imaging using resting-state fluctuations. *Neuroimage*, *7*(2), 119–132. 10.1006/nimg.1997.0315. 9558644 10.1006/nimg.1997.0315

[CR18] Menon, V. (2011). Large-scale brain networks and psychopathology: A unifying triple network model. *Trends in Cognitive Sciences*, *15*(10), 483–506. 10.1016/j.tics.2011.08.003. 21908230 10.1016/j.tics.2011.08.003

[CR19] Nellessen, N., Rottschy, C., Eickhoff, S. B., Ketteler, S. T., Kuhn, H., Shah, N. J., Schulz, J. B., Reske, M., & Reetz, K. (2015). Specific and disease stage-dependent episodic memory-related brain activation patterns in Alzheimer’s disease: A coordinate-based meta-analysis. *Brain Struct Funct*, *220*(3), 1555–1571. 10.1007/s00429-014-0744-6. 24633738 10.1007/s00429-014-0744-6 PMC8004564

[CR20] Patel, G. H., Yang, D., Jamerson, E. C., Snyder, L. H., Corbetta, M., & Ferrera, V. P. (2015). Functional evolution of new and expanded attention networks in humans. *Proc Natl Acad Sci U S A*, *112*(30), 9454–9459. 10.1073/pnas.1420395112. 26170314 10.1073/pnas.1420395112 PMC4522817

[CR22] Petersen, R. C., Stevens, J. C., Ganguli, M., Tangalos, E. G., Cummings, J. L., & Dekosky, S. T. (2001). Practice parameter: Early detection of dementia: Mild cognitive impairment (an evidence-based review). *Report of the Quality Standards Subcommittee of the American Academy of Neurology Neurology*, *56*(9), 1133–1142. 10.1212/wnl.56.9.1133. 10.1212/wnl.56.9.1133 11342677

[CR21] Petersen, R. C., Lopez, O., Armstrong, M. J., Getchius, T., Ganguli, M., Gloss, D., Gronseth, G. S., Marson, D., Pringsheim, T., Day, G. S., Sager, M., Stevens, J., & Rae-Grant, A. (2018). Practice guideline update summary: Mild cognitive impairment: Report of the Guideline Development, Dissemination, and implementation Subcommittee of the American. *Academy of Neurology Neurology*, *90*(3), 126–135. 10.1212/WNL.0000000000004826. 29282327 10.1212/WNL.0000000000004826 PMC5772157

[CR23] Power, J. D., Barnes, K. A., Snyder, A. Z., Schlaggar, B. L., & Petersen, S. E. (2012). Spurious but systematic correlations in functional connectivity MRI networks arise from subject motion. *Neuroimage*, *59*(3), 2142–2154. 10.1016/j.neuroimage.2011.10.018. 22019881 10.1016/j.neuroimage.2011.10.018 PMC3254728

[CR24] Qi, Z., Wu, X., Wang, Z., Zhang, N., Dong, H., Yao, L., & Li, K. (2010). Impairment and compensation coexist in amnestic MCI default mode network. *Neuroimage*, *50*(1), 48–55. 10.1016/j.neuroimage.2009.12.025. 20006713 10.1016/j.neuroimage.2009.12.025

[CR25] Saad, Z. S., Reynolds, R. C., Jo, H. J., Gotts, S. J., Chen, G., Martin, A., & Cox, R. W. (2013). Correcting brain-wide correlation differences in resting-state FMRI. *Brain Connectivity*, *3*(4), 339–352. 10.1089/brain.2013.0156. 23705677 10.1089/brain.2013.0156 PMC3749702

[CR26] Scheltens, P., Leys, D., Barkhof, F., Huglo, D., Weinstein, H. C., Vermersch, P., Kuiper, M., Steinling, M., Wolters, E. C., & Valk, J. (1992). Atrophy of medial temporal lobes on MRI in probable Alzheimer’s disease and normal ageing: Diagnostic value and neuropsychological correlates. *Journal of Neurology, Neurosurgery and Psychiatry*, *55*(10), 967–972. 10.1136/jnnp.55.10.967. 1431963 10.1136/jnnp.55.10.967 PMC1015202

[CR27] Seo, E. H., & Choo, I. L. (2016). Amyloid-independent functional neural correlates of episodic memory in amnestic mild cognitive impairment. *European Journal of Nuclear Medicine and Molecular Imaging*, *43*(6), 1088–1095. 10.1007/s00259-015-3261-9. 26613793 10.1007/s00259-015-3261-9

[CR28] Taylor, K. S., Seminowicz, D. A., & Davis, K. D. (2009). Two systems of resting state connectivity between the insula and cingulate cortex. *Human Brain Mapping*, *30*(9), 2731–2745. 10.1002/hbm.20705. 19072897 10.1002/hbm.20705 PMC6871122

[CR29] Touroutoglou, A., Hollenbeck, M., Dickerson, B. C., & Feldman, B. L. (2012). Dissociable large-scale networks anchored in the right anterior insula subserve affective experience and attention. *Neuroimage*, *60*(4), 1947–1958. 10.1016/j.neuroimage.2012.02.012. 22361166 10.1016/j.neuroimage.2012.02.012 PMC3345941

[CR30] Wang, L., Zang, Y., He, Y., Liang, M., Zhang, X., Tian, L., Wu, T., Jiang, T., & Li, K. (2006). Changes in hippocampal connectivity in the early stages of Alzheimer’s disease: Evidence from resting state fMRI. *Neuroimage*, *31*(2), 496–504. 10.1016/j.neuroimage.2005.12.033. 16473024 10.1016/j.neuroimage.2005.12.033

[CR33] Wang, Z., Xia, M., Dai, Z., Liang, X., Song, H., He, Y., & Li, K. (2015). Differentially disrupted functional connectivity of the subregions of the inferior parietal lobule in Alzheimer’s disease. *Brain Struct Funct*, *220*(2), 745–762. 10.1007/s00429-013-0681-9. 24292325 10.1007/s00429-013-0681-9

[CR31] Wang, P., Li, R., Yu, J., Huang, Z., Yan, Z., Zhao, K., & Li, J. (2017). Altered distant synchronization of background network in mild cognitive impairment during an executive function Task. *Frontiers in Behavioral Neuroscience*, *11*, 174. 10.3389/fnbeh.2017.00174. 29018338 10.3389/fnbeh.2017.00174 PMC5614929

[CR32] Wang, S., Sun, H., Hu, G., Xue, C., Qi, W., Rao, J., Zhang, F., Zhang, X., & Chen, J. (2021). Altered Insular Subregional Connectivity Associated with cognitions for distinguishing the spectrum of pre-clinical Alzheimer’s Disease. *Frontiers in Aging Neuroscience*, *13*, 597455. 10.3389/fnagi.2021.597455. 33643021 10.3389/fnagi.2021.597455 PMC7902797

[CR34] Whitfield-Gabrieli, S., & Nieto-Castanon, A. (2012). Conn: A functional connectivity toolbox for correlated and anticorrelated brain networks. *Brain Connectivity*, *2*(3), 125–141. 10.1089/brain.2012.0073. 22642651 10.1089/brain.2012.0073

[CR35] Wolk, D. A., & Klunk, W. (2009). Update on amyloid imaging: From healthy aging to Alzheimer’s disease. *Current Neurology and Neuroscience Reports*, *9*(5), 345–352. 10.1007/s11910-009-0051-4. 19664363 10.1007/s11910-009-0051-4 PMC2825106

[CR37] Xie, C., Goveas, J., Wu, Z., Li, W., Chen, G., Franczak, M., Antuono, P. G., Jones, J. L., Zhang, Z., & Li, S. J. (2012b). Neural basis of the association between depressive symptoms and memory deficits in nondemented subjects: Resting-state fMRI study. *Human Brain Mapping*, *33*(6), 1352–1363. 10.1002/hbm.21291. 21618660 10.1002/hbm.21291 PMC3190573

[CR36] Xie, C., Bai, F., Yu, H., Shi, Y., Yuan, Y., Chen, G., Li, W., Chen, G., Zhang, Z., & Li, S. J. (2012a). Abnormal insula functional network is associated with episodic memory decline in amnestic mild cognitive impairment. *Neuroimage*, *63*(1), 320–327. 10.1016/j.neuroimage.2012.06.062. 22776459 10.1016/j.neuroimage.2012.06.062 PMC4513936

[CR38] Zanchi, D., Montandon, M. L., Sinanaj, I., Rodriguez, C., Depoorter, A., Herrmann, F. R., Borgwardt, S., Giannakopoulos, P., & Haller, S. (2017). Decreased Fronto-Parietal and increased default Mode Network activation is Associated with subtle cognitive deficits in Elderly Controls. *Neurosignals*, *25*(1), 127–138. 10.1159/000486152. 29268260 10.1159/000486152

[CR40] Zhang, S., & Li, C. S. (2012). Functional connectivity mapping of the human precuneus by resting state fMRI. *Neuroimage*, *59*(4), 3548–3562. 10.1016/j.neuroimage.2011.11.023. 22116037 10.1016/j.neuroimage.2011.11.023 PMC3288461

[CR39] Zhang, J., Wang, J., Xu, X., You, Z., Huang, Q., Huang, Y., Guo, Q., Guan, Y., Zhao, J., Liu, J., Xu, W., Deng, Y., Xie, F., & Li, B. (2023). In vivo synaptic density loss correlates with impaired functional and related structural connectivity in Alzheimer’s disease. *Journal of Cerebral Blood Flow and Metabolism*, *43*(6), 977–988. 10.1177/0271678X231153730. 36718002 10.1177/0271678X231153730 PMC10196742

[CR41] Zhou, J., Greicius, M. D., Gennatas, E. D., Growdon, M. E., Jang, J. Y., Rabinovici, G. D., Kramer, J. H., Weiner, M., Miller, B. L., & Seeley, W. W. (2010). Divergent network connectivity changes in behavioural variant frontotemporal dementia and Alzheimer’s disease. *Brain*, *133*(Pt 5), 1352–1367. 10.1093/brain/awq075. 20410145 10.1093/brain/awq075 PMC2912696

