# Supplementary material for: Increased anterior insula connectivity associated with cognitive maintenance in amnestic mild cognitive impairment: a longitudinal study
Source: Brain Imaging Behav. 2024 May 24;18(5):1001–9. doi: 10.1007/s11682-024-00899-2 (PMC11582194; doi:10.1007/s11682-024-00899-2)
Supplement: Supplementary file 1 — Supplementary Material 1 [file 11682_2024_899_MOESM1_ESM.docx]

**Supplementary materials**

**Figure 1.** Whole brain functional connectivity of left anterior insular within aMCI and HCs for baseline and follow-up respectively. Numbers in the figure indicate the *Z* coordinate in Montreal Neurological Institute.

**
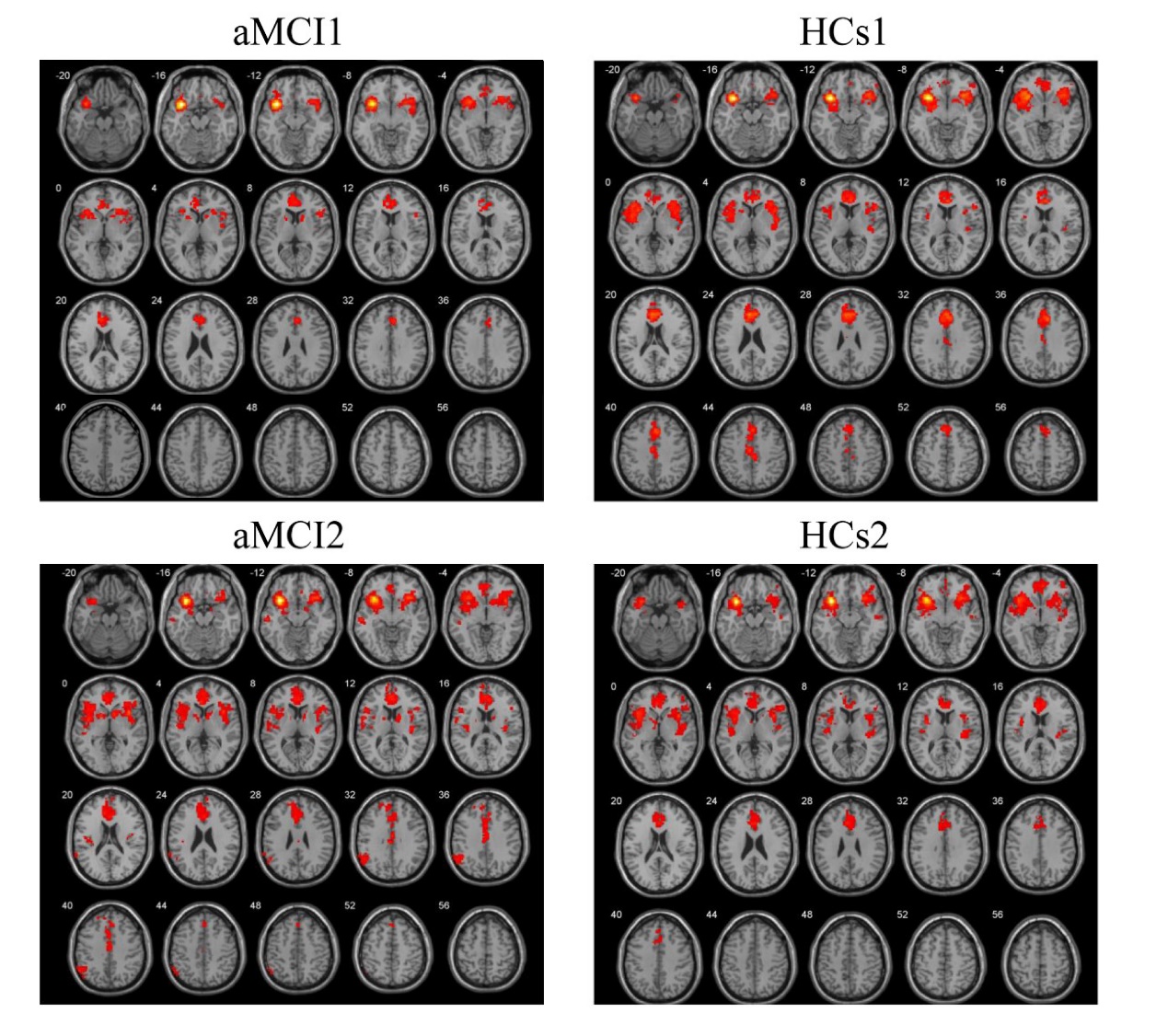
**

**
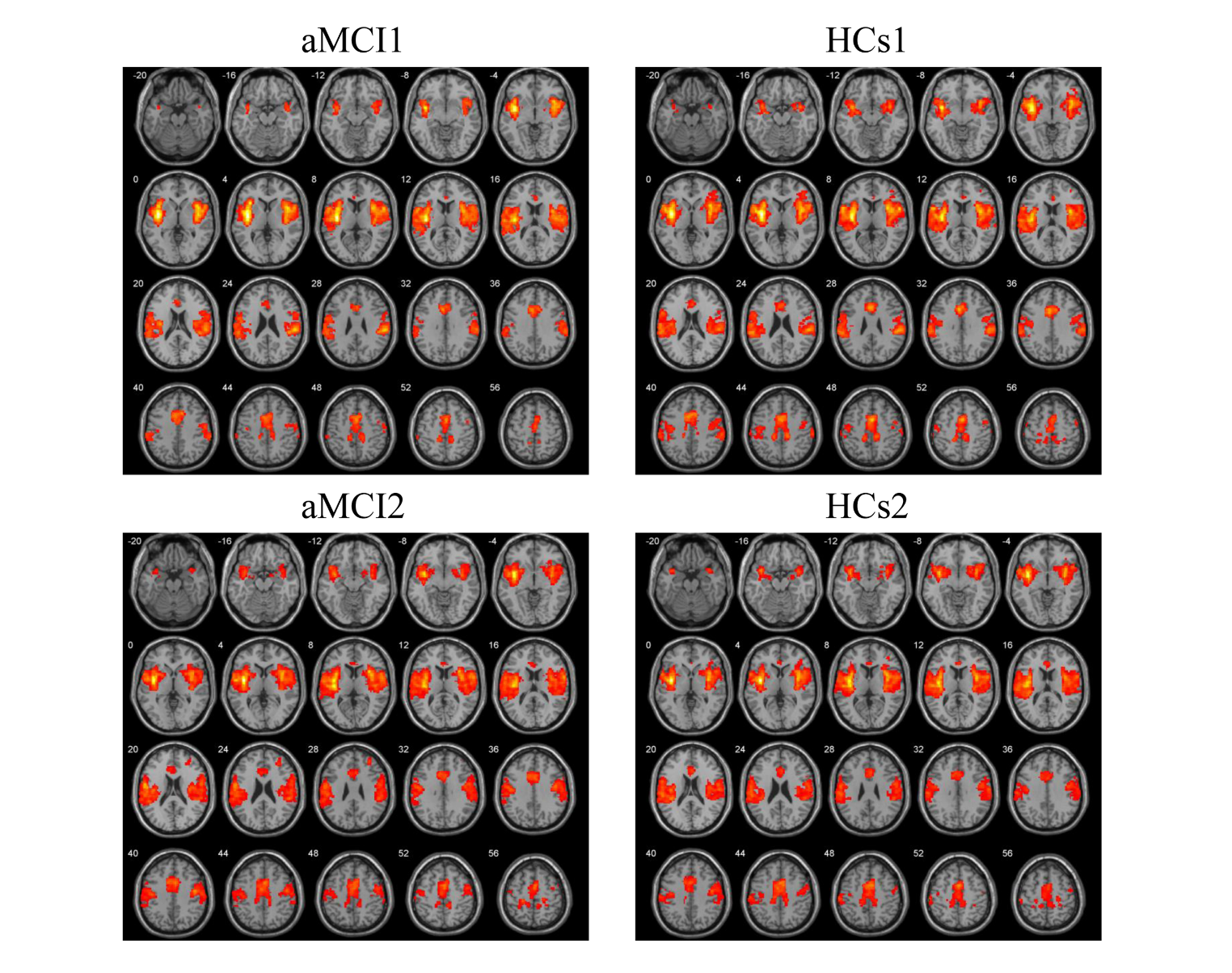
Figure 2.** Whole brain functional connectivity of left posterior insular within aMCI and HCs for baseline and follow-up respectively. Numbers in the figure indicate the *Z* coordinate in Montreal Neurological Institute.


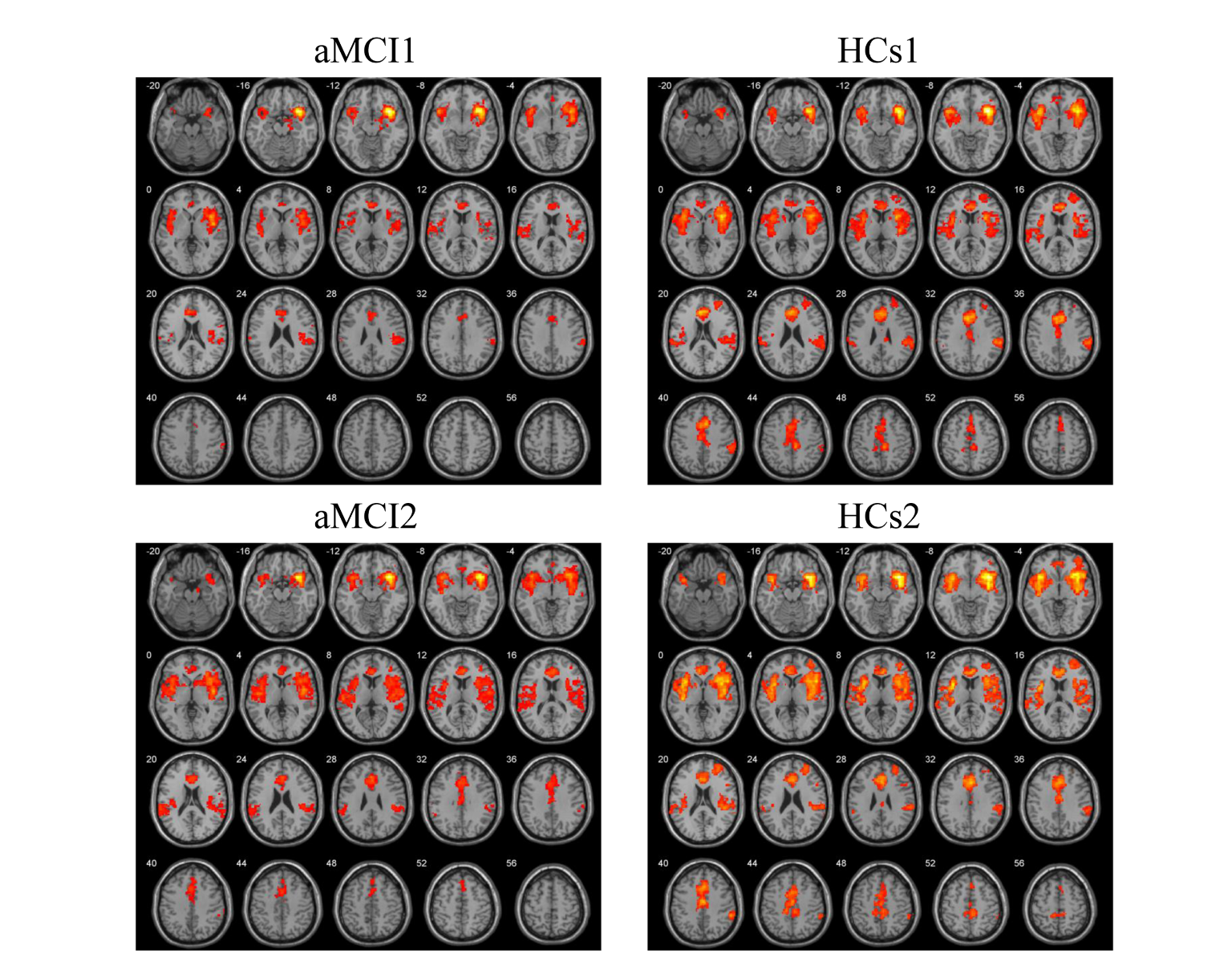
**Figure 3**. Whole brain functional connectivity of right anterior insular within aMCI and HCs for baseline and follow-up respectively. Numbers in the figure indicate the *Z* coordinate in Montreal Neurological Institute.


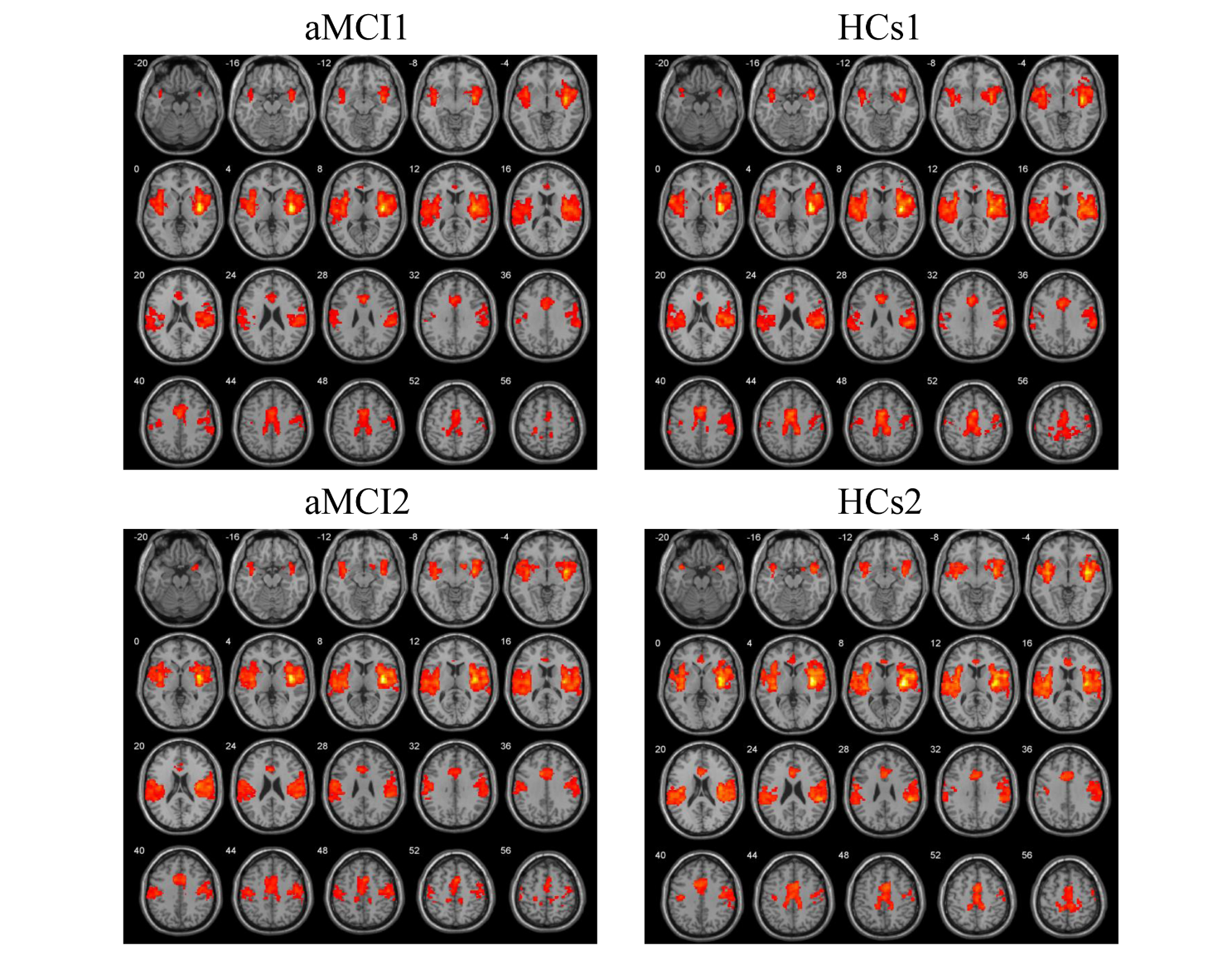
**Figure 4**. Whole brain functional connectivity of right posterior insular within aMCI and HCs for baseline and follow-up respectively. Numbers in the figure indicate the Z coordinate in Montreal Neurological Institute.
